# Macroecological processes impact Australian soil resistomes and climatically stable regions with anthropogenic activities serve as ARG hotspots

**DOI:** 10.1093/ismejo/wrag079

**Published:** 2026-04-10

**Authors:** Mingming Du, Peipei Xue, Budiman Minasny, Ho J Jang, Alex McBratney

**Affiliations:** School of Life and Environmental Sciences, The University of Sydney, Sydney, New South Wales 2015, Australia; School of Life and Environmental Sciences, The University of Sydney, Sydney, New South Wales 2015, Australia; School of Life and Environmental Sciences, The University of Sydney, Sydney, New South Wales 2015, Australia; School of Life and Environmental Sciences, The University of Sydney, Sydney, New South Wales 2015, Australia; School of Life and Environmental Sciences, The University of Sydney, Sydney, New South Wales 2015, Australia

**Keywords:** antibiotic resistance genes, climatic variability, land-use intensification, soil resistome, spatial modelling

## Abstract

Soil antibiotic resistance genes (ARGs) pose a global health threat, but a critical knowledge gap remains regarding how macro-scale pedoclimatic constraints interact with land-use intensification to determine the spatial distribution of the soil resistome. To address this, we conducted a continental-scale survey of Australian topsoils and used metagenomic analysis to reveal the hierarchy of drivers shaping soil resistome. Machine learning was applied to predict the spatial ARG distribution across Australia. We found that, at the continental scale, climatic variability acts as the dominant filter on ARG distribution, overriding local soil properties and human disturbance. Unexpectedly, climatically stable regions, characterized by sandy and low-carbon soils in Southwestern Australia, emerged as ARG hotspots. We also demonstrated that anthropogenic land use amplifies ARG abundance within these climatically stable regions. Furthermore, spatial modelling revealed distinct geographical patterns: although total ARG abundance was enriched in coastal regions, specific resistance mechanisms showed unique distributions. As a continental-scale investigation of soil ARGs in Australia, this study provides a framework to identify high-risk regions where lower climatic variability and intensive farming interact to enhance antimicrobial resistance.

## Introduction

Antimicrobial resistance (AMR) constitutes a critical global health crisis threatening human health and imposing substantial economic burdens worldwide [1, 2], which is recognized as one of the major challenges in the “One Health” framework. The “One Health” approach recognizes that human health is linked to the health of animals, plants, and the broader environment [3]. Central to this is the ubiquity of Antibiotic Resistance Genes (ARGs) across diverse environments, including water, sediments, and soils [4]. As the Earth’s most biodiverse habitat, soil serves as a crucial reservoir and conduit for the horizontal transfer of resistance mechanisms among microorganisms [5]. Many soil-borne resistance genes exhibit high sequence homology with those identified in human pathogens [6]. Consequently, understanding the drivers of the spatial distribution of soil ARGs is crucial for predicting resistance hotspots and developing effective pollution control strategies.

Global-scale initiatives have begun to map the soil resistome, identifying climatic factors, edaphic properties, and human activities as key drivers [7, 8]. However, a critical knowledge gap remains regarding the relative importance of these drivers. Global studies may underrepresent the fine-scale heterogeneity, obscuring how macro-scale climatic constraints interact with local conditions to shape resistance profiles. This lack of resolution limits their utility for regional risk assessment. This is particularly true for Australia, a key agricultural region that remains poorly represented in global data [7]. Consequently, we lack baseline ARG profiles for most Australian soil types and have a limited understanding of how climate variability, soil types, and land-use change influence ARG abundance. This knowledge gap not only hinders the World Health Organization (WHO) Global Action Plan in identifying priority regions for combating soil antibiotic resistance but also complicates the assessment of health risks [9].

To address these gaps, we analyzed topsoil samples from a continental-scale survey across Australia. We focused on the regions within the 500–600 mm isohyet with the most intensive agricultural activities in Australia to specifically assess how human interventions influence the soil resistome. Unlike previous studies that mostly relied on amplification-based methods [10], we employed shotgun metagenomics to capture the full diversity of the resistome. We generated a comprehensive metagenomic dataset comprising 325 ARGs from 268 soil samples and integrated these data with detailed pedo-climatic and land-use information. We hypothesized that (1) macro-scale climate variability explains more variation in the soil resistome than edaphic properties; (2) the effects of land-use intensification are habitat-dependent; and (3) ARG abundance and dominant resistance mechanisms exhibit spatially structured, predictable patterns across Australia. These advances provide a habitat-based, climate-aware framework for identifying antibiotic resistance hotspots and supporting evidence-based land management strategies.

## Materials and methods

### Study design

Soil samples were collected within the 500–600 mm annual rainfall zone in Australia, where most agricultural activity clusters, allowing us to capture the influence of land-use intensity on ARG distribution (Fig. S1). First, we established 65 sampling buffers spaced roughly 100 km apart (Fig. S1a). Within these buffers, we targeted the dominant soil types and ensured the collection of paired land-use samples (natural and agricultural) located within 5 km of each other. This transect captured a climatic range where mean annual temperature (MAT) ranged from 14°C to 28°C, and mean annual precipitation (MAP) was constrained within 500–600 mm. At each site, topsoil samples (0–10 cm) were collected with three replicates. Due to challenges in sampling in remote areas and the lack of appropriate natural soil at some locations, our final sampling design was adjusted. In total, we collected 268 soil samples from 55 buffers: 107 from natural sites (Fig. S1b) and 161 from agricultural sites (Fig. S1c), the latter comprising 95 from pasture soils and 66 from cropland. Natural samples were collected from native vegetation sites that had not experienced agricultural activity in the past 10 years, including forests, woodlands, and bushlands. Pasture samples were obtained from grasslands used for grazing, and cropland samples were collected from fields under crop production, including wheat, canola, barley, alfalfa, and cotton. After collection, each sample was divided into two subsamples: one was air-dried for physicochemical analysis, and the other was stored at −20°C prior to DNA extraction.

### Soil physicochemical characterization and climatic data acquisition

We measured soil pH and soil texture following previous analysis [11]. Briefly, soil texture were quantified using the hydrometer method; soil pH was measured in a 0.01 M CaCl₂ suspension (1:5 w/v). Other essential soil properties, including total carbon (TC), total phosphorus (TP), total sulphur (TS), total nitrogen (TN), soil organic carbon (SOC), and carbon to nitrogen ratio, were determined using standard methods [12].

Climate variables including mean annual precipitation (MAP), mean annual temperature (MAT), aridity index (AI), actual evapotranspiration (AET), annual precipitation variability (PVA), annual temperature range (TRA), and annual atmospheric water deficit (WDA), were extracted based on our sampling coordinates from TERN datasets using the “SLGACloud” package in R [13]. Detailed pedo-climatic factors along the continent sampling sites are provided (Fig. S2).

### Metagenomic analysis and ARG annotation

Total soil microbial DNA was extracted from 0.25 g of soil using the Qiagen DNeasy PowerSoil Pro Kit following the manufacturer’s instructions. Following quality control, the purified DNA was fragmented to prepare the libraries and then sequenced on a NovaSeq 6000 System (Illumina) using a 150-bp paired-end strategy. Approximately 20 Gbp of sequencing data were generated per sample.

The raw metagenomic sequencing was filtered and primer trimmed using Trimmomatic [14]. After quality control, approximately 100–200 million clean reads remained per sample (Fig. S3). These clean reads then underwent de novo metagenomic assembly using MEGAHIT by K-mers with k-min set at 35–95 and a step size of 20 to generate initial scaffolds [15]. After fragmenting the scaffolds, only scaftigs exceeding 500 bp were retained for downstream analysis. The open reading frames (ORFs) were predicted using Prodigal from the filtered scaftigs, and any ORF shorter than 90 bp was removed [16].

To create a non-redundant gene catalogue, the predicted ORFs were proceeded using Mmseqs software with default parameters for clustering and redundancy removel [17]. For each resulting cluster, the longest sequence was selected as the representative sequence (unigene), generating our final non-redundant gene catalogue. After confirming quality, clean reads were aligned to this catalogue using BBMap [18]. For the annotation of Antibiotic Resistance Genes (ARGs), the unigene catalogue was blasted against the Comprehensive Antibiotic Resistance Database (CARD) using Diamond [19]. To identify the potential microbial hosts of these ARGs, the ARG-carrying reads were taxonomically assigned by searching them against the NCBI non-redundant protein sequence database (NR) [20]. The selection criteria of E-value <1e – 4, identity >80% were used for the ARG gene annotation. Finally, the abundance of each ARG in each sample was quantified as Transcripts Per Million (TPM) [21].

### XGBoost machine learning model

We employed an XGBoost model to evaluate the importance of potential environmental drivers influencing ARG abundance, utilizing the “xgboost” package in R [22]. Our analysis included 17 candidate predictors: alpha diversity (Shannon Index) and a range of edaphic and climatic variables. Prior to analysis, we split the data into a training set (70%) and a hold-out test set (30%). Model selection focused specifically on determining the optimal number of boosting rounds (best_nrounds), keeping other hyperparameters fixed a priori to balance model bias and variance. We performed a 5-fold cross-validation on the training set, using an upper limit of 2000 rounds and early stopping (after 50 rounds without improvement). The iteration that generated the lowest mean RMSE was selected as best_nrounds. The final model was then refit on the full training set using this optimal number of rounds. To interpret the functional form and overall impact of each feature, we computed SHapley Additive explanations (SHAP) values using the trained XGBoost model and the training predictors. Global feature importance was derived as the mean absolute SHAP value per feature. These results were visualized using a beeswarm plot. Finally, SHAP dependence plots were generated to thoroughly characterize the functional form of each predictor’s effect on ARGs.

### K-means clustering

To simplify and delineate the environmental gradient, K-means clustering was applied to cluster pedo-climatic habitats [23], based on two key features identified from our XGBoost and PLS-PM results: clay content (representing soil properties) and PVA (representing climatic variation). Prior to clustering, these two variables were extracted and standardized (using Z-scores) to ensure equal weighting. The optimal number of clusters (K) was assessed visually and programmatically using the Gap Statistic method (K.max =10, B = 50, nstart = 25). The final K-means model was run based on the Gap statistic method results. For interpretability, we visualised cluster separation using the original (unscaled) values. This analysis was implemented using the “cluster” package in R.

### PLS-PM

We used partial least-squares path modelling (PLS-PM) to quantify the direct and indirect effects of land-use intensity and climatic variation on the abundance of ARGs, using the “plspm” package in R [24]. Variables were selected based on our SHAP value results. PVA and TRA were defined as climate variation; TP, TS, SOC, and C:N ratio were defined as soil resources; clay and sand content were defined as soil texture; land-use intensity was represented by the observed land-use classes (dummy-coded) forming the land use construct. The bacterial host Shannon Index represented the bacterial diversity. To limit indicator bias, items with outer loadings <0.70 were treated as candidates for removal, as loadings ≥0.70 indicate that at least ~50% of an indicator’s variance is captured by the latent construct.

### Digital mapping

We utilized the Quantile Random Forest (QRF) [25] model to develop regression models for estimating ARG abundance across Australia [26]. The model incorporated a comprehensive suite of environmental variables, including soil properties, climatic factors, vegetation cover, and relief, which were sourced from 90 m resolution raster maps via the “SLGACloud” R package (Table S1). To ensure reproducibility and consistency throughout the modelling process, we fixed random seeds before cross-validation (CV) and tuning, maintained a consistent projected Coordinate Reference System (CRS) between point and raster data.

The QRF model was trained and evaluated using a spatial block cross-validation scheme (10 folds), with 200-km blocks randomly assigned to folds; the random allocation was repeated 10 times to improve fold balance. In each fold, the model was trained on nine folds and evaluated on the held-out fold. Random forest hyperparameters were set to default value (mtry = 4; min.node.size = 5; num.trees = 250). We assessed the model performance based on the assembled cross-validated predictions by calculating the mean error (ME), root mean squared error (RMSE), coefficient of determination (*R*^2^) and concordance correlation (ρc) (Table S2). The RMSE describes how large the prediction errors are on average and *R*^2^ describes how well the model reproduces variability in the observed data. Following cross-validation, we trained a final Random Forest model on the full dataset. Variable importance was then evaluated using the impurity approach for model interpretation [27]. Finally, the trained model was applied to the co-registered covariate raster stack to generate 1 km resolution prediction maps for both the ARG abundance and its allocated mechanisms across Australia.

### Statistical analysis

To evaluate spatial patterns, distance-decay relationships (DDRs) were constructed to assess the correlation between geographic distance and ARG community dissimilarity, with the significance assessed using the Mantel test by the “vegan” package. Sampling sites and associated environmental data were visualized using the “sf,” “rnaturalearth,” “terra,” and “ggplot2” R packages. We further characterized community attributes by calculating Shannon index to represent the alpha diversity of potential hosts using the “vegan” package. Based on feature importance rankings from prior XGBoost models, linear regressions were employed to visualize the relationships between key environmental drivers and ARG abundance. To investigate the impact of land use, variations in ARG abundance and community composition were evaluated using one-way ANOVA and Permutational Multivariate Analysis of Variance (PERMANOVA) based on Bray–Curtis dissimilarity metric, respectively. Furthermore, community composition across various land-use types and habitats was visualized using stacked bar charts generated with the “ggplot2” package. The key R packages and their versions used for these statistical analyses are provided (Table S3).

## Results

### Climatic–edaphic habitats structure the baseline distribution of ARGs in Australian soils

**Figure 1:**
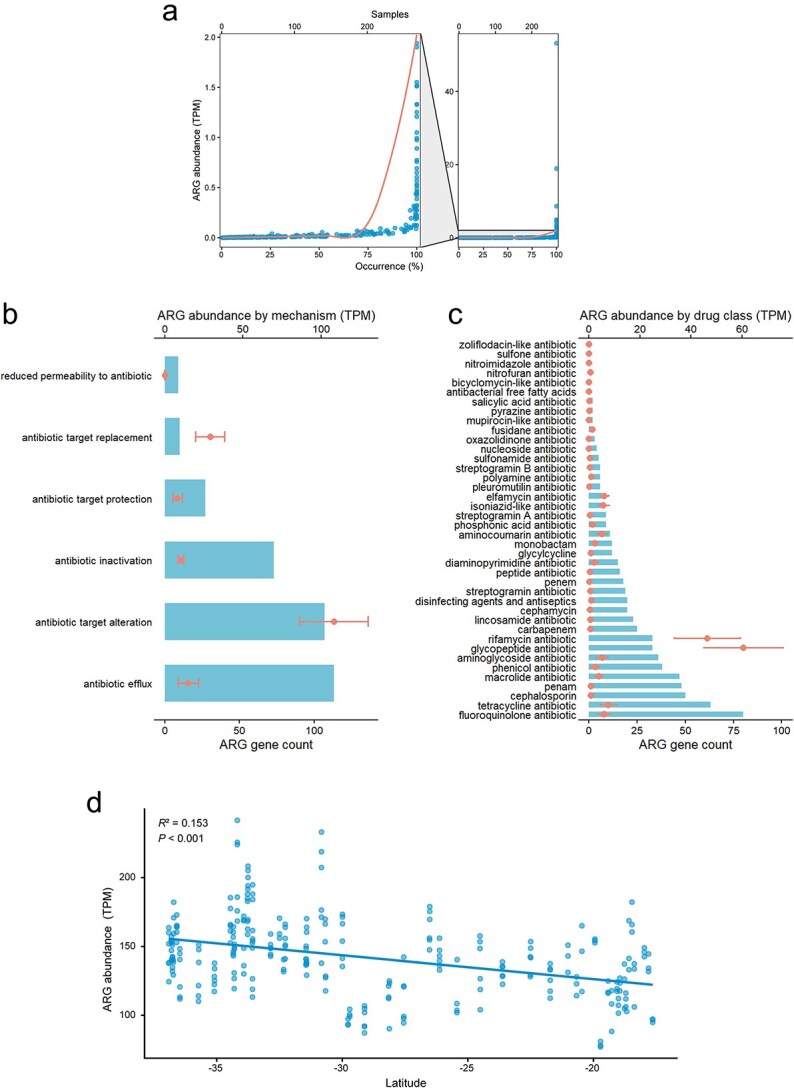
Distribution of ARGs across Australia. (a) ARG occurrence and abundance. The bottom x-axis shows the occurrence (%) of each gene, and the top x-axis shows the number of samples containing each gene. (b) The abundance of ARGs for each resistance mechanism. Bars denote the total ARG gene count (bottom x-axis), and points indicate the mean abundance (±SD) (top x-axis). (c) The abundance of ARGs for each resistance drug class. Bars denote the total ARG gene count (bottom x-axis), and points indicate the mean abundance (±SD) (top x-axis). (d) Relationship between ARG abundance and latitude.

After processing the metagenomic data from the 268 topsoil samples, we detected 325 antimicrobial-resistance genes (ARGs) spanning 6 resistance mechanisms and 40 drug classes (Fig. 1). The ARG distribution was widespreaded in Australia, with 13.8% of ARGs occurring in all the samples (Fig. 1a). Those ARGs were classified into 6 groups based on the resistance mechanism (Fig. 1b). Amongst them, antibiotic efflux (113 associated genera) and antibiotic inactivation (107 associated genera) were the two most diverse in terms of the number of associated genera. Conversely, although not the most diverse, antibiotic target alteration showed the highest abundance across samples (TPM = 108), followed by antibiotic target replacement (TPM = 29). Regarding the drug class of these ARGs (Fig. 1c), fluoroquinolone and tetracycline antibiotic classes ranked as the top two in terms of total gene count. However, shifting the focus to the abundance, the glycopeptide (TPM = 60.4) and rifamycin (TPM = 46.2) antibiotic classes emerged as the most dominant.

Spatially, total ARG abundance exhibited a significant latitudinal gradient, decreasing from higher latitudes (TPM = 158) to lower latitudes (TPM = 96) (Fig. 1d). This spatial decline was associated with specific climatic shifts. Towards the lower latitudes, MAT and PVA increased, whileAI and WDA decreased. This linear climatic association contrasts with the non-monotonic patterns of some soil properties (e.g. pH, clay), which peaked in mid-latitudes (Fig. S2).

### Climatic variability and soil properties control the continental-scale ARG distribution

**Figure 2:**
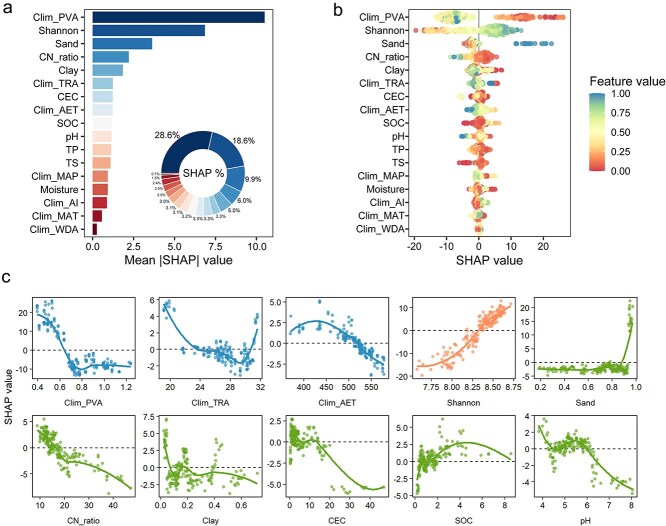
Environmental drivers of soil ARG abundance across Australia revealed by SHAP interpretation model. (a) Mean absolute SHAP ranking of predictors; inset shows relative contribution (%). (b) The SHAP beeswarm showing per-sample effects of each predictor (points coloured by normalized feature value). (c) SHAP dependence plots for the top ten predictors. Curves are loess smooths; dashed line marks zero effect. TP: Total phosphorus; TS: Total sulphur; CEC: Cation exchange capacity; CN_ratio: Carbon-to-nitrogen ratio; SOC: Soil organic carbon. Clim_MAT: Mean annual temperature; Clim_MAP: Mean annual precipitation; Clim_AI: Aridity index; Clim_AET: Annual actual evapotranspiration; Clim_PVA: Annual precipitation variability; Clim_TRA: Annual temperature range; Clim_WDA: Annual atmospheric water deficit.

Having established that broad climatic gradients and edaphic conditions significantly shaped ARG community similarity (Fig. S4a), we next sought to identify the primary drivers of ARG abundance. To do so, we fitted an XGBoost model incorporating all candidate pedo-climatic variables and quantified their relative contributions using SHAP values (Fig. 2). Climatic variability emerged as the dominant driver, with annual precipitation variability (PVA) contributing 28.6% of the total importance, followed by bacterial Shannon Index (18.6%), and sand content (9.9%) (Fig. 2a).

SHAP beeswarm and dependence plots elucidated the non-linear effects of environmental drivers and identified critical thresholds (Fig. 2b and c). For instance, higher ARG abundance was associated with lower precipitation variability (typically <0.65) and annual temperature range (value <21). Conversely, higher bacterial Shannon Index (>8.3) was also consistently linked to elevated ARG abundance. Soil physicochemical properties modulated the strength of this climatic filtering: ARG abundance tended to be lower in fine-textured soils (Fig. 2c). These patterns were broadly consistent with the linear relationships between individual predictors and ARG abundance (Fig. S4b).

### Land-use intensification amplifies ARG abundance selectively within specific pedo-climatic habitats

**Figure 3:**
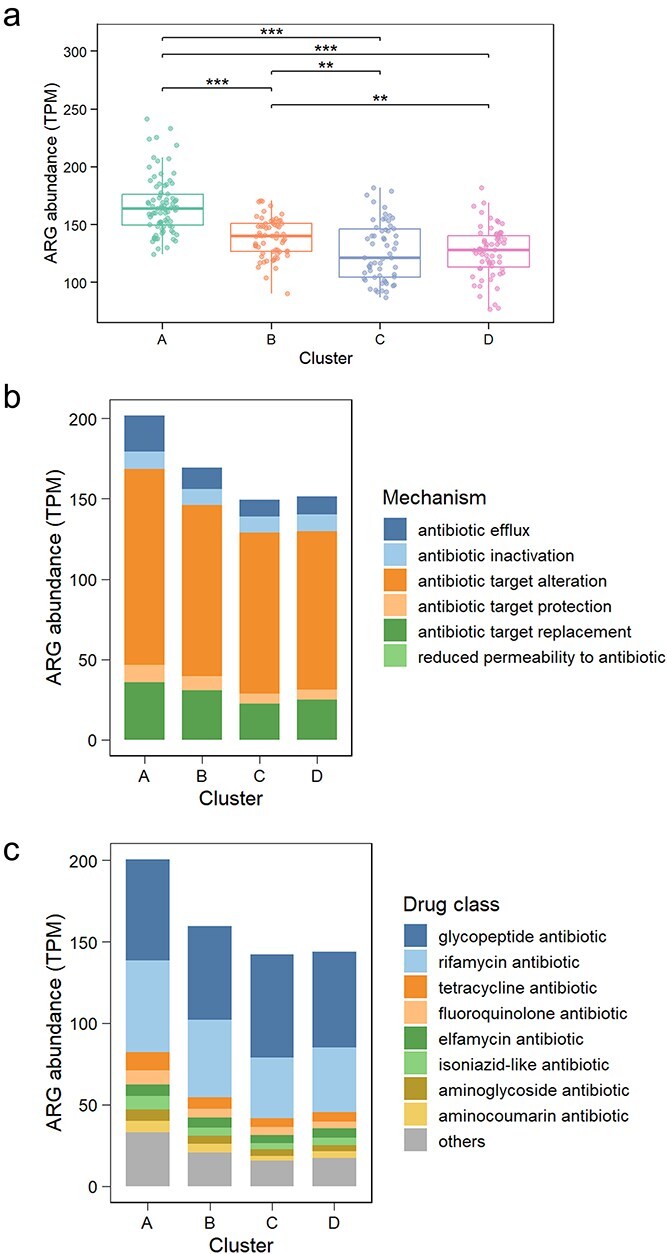
ARG distribution across different pedo-climatic clusters. (a) Total ARG abundance for each pedo-climatic cluster. (b) ARG abundance partitioned by resistance mechanism. (c) ARG abundance partitioned by drug class. Significance levels in panel A are as follows: ^*^P < .05; ^**^P < .01; and ^***^P < .001.

Given that land use was not a primary predictor at the continental scale, we applied the K-means clustering to investigate distinct pedo-climatic habitats (Fig. S5a and b). The identified four clusters could be assigned to two main habitat types: climatically stable, coarse-textured soils (Clusters A and B, in Southwest and Southeast Australia) versus more variable, medium-fine texture habitats (Clusters C and D, in Eastern seaboard and Northern Australia) (Fig. S5c). Cluster C was characterized by the highest soil pH, Cluster D by the greatest precipitation variability, Cluster B by the relatively lower soil pH and precipitation variability, and Cluster A by the lowest precipitation variability and clay (Fig. S6). This environmental partitioning translated into a clear latitudinal gradient, with Clusters A and B occurring predominantly at higher latitudes and Clusters C and D progressively closer to lower latitudes (Fig. S5d). We further found that ARG abundance in climatically stable, coarse-textured habitats (Clusters A and B) was higher than in more climatically variable habitats with medium-to-fine textures (Clusters C and D) (Fig. 3a). Across these habitats, differences in ARG composition were mainly driven by shifts in resistance mechanisms, particularly genes encoding antibiotic target alteration and target replacement (Fig. 3b), and at the drug-class level were largely associated with rifamycin resistance genes (Fig. 3c).

**Figure 4:**
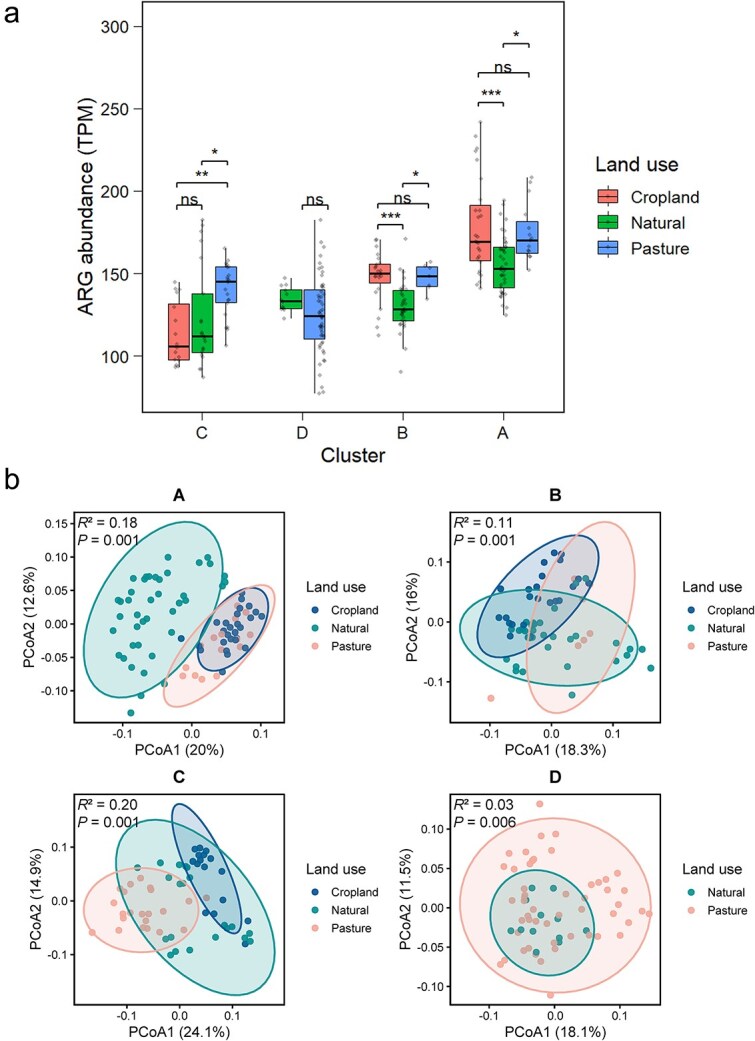
Influence of land use intensity on ARG abundance and community composition across different pedo-climatic clusters. (a) The abundance of soil ARGs (TPM) compared by land use for each pedo-climatic cluster. Within each cluster, differences amongst land-use types were first tested using one-way ANOVA, followed by Tukey’s HSD post hoc test. The overall ANOVA results were Cluster C, *F* = 6.7, *P* = .003; Cluster D, *F* = 2.0, *P* = .167; Cluster B, *F* = 11.6, *P* < .001; and Cluster A, *F* = 10.4, *P* < .001. (b) ARG community composition by land use within each cluster, visualised by PCoA based on Bray–Curtis distances. Significance levels in panel A are as follows: ns, not significant; ^*^P < .05; ^**^P < .01; and ^***^P < .001.

The impact of land use on ARGs was strongly context-dependent (Fig. 4). In climatically more stable, coarse-textured habitats (Clusters A and B, in Southwest and Southeast Australia), cropland and pasture consistently elevated ARG abundance by 13.5% compared to natural sites. This increase was not uniform across mechanisms: land-use intensification selectively enhanced genes involved in antibiotic target alteration by 11.4%, including rifamycin resistance, rather than proportionally increasing all ARG classes (Fig. S7). By contrast, the effects in the more variable or medium-fine texture habitats (Clusters C and D, in Eastern seaboard and Northern Australia) were bidirectional rather than consistently positive. Under pasture, ARG abundance decreased significantly by 6.1% in Cluster D (in Northern Australia), whereas it increased by 16.4% in Cluster C (in the Eastern seaboard). Under cropland, ARG abundance decreased by 7.7% in Cluster C. These fluctuations, whether positive or negative, were primarily driven by shifts in genes encoding antibiotic target alteration (Fig. S7). Finally, driver analysis within each habitat indicated that soil properties explained more variation in ARG patterns than climatic factors (Fig. S8).

The PLS-PM analysis indicated that ARG abundance is regulated by climatic variability and land-use intensity (Fig. S9). Climatic variation, constructed from annual precipitation variability (PVA) and annual temperature range (TRA), exerted a strong negative direct effect on ARG abundance (direct effect = −0.51, *P* < .001). In contrast, land-use intensity showed a significant positive direct effect on ARG abundance (direct effect = 0.11, *P* = .021). Beyond these direct effects, both climatic variation and land-use intensity influenced ARG abundance indirectly via bacterial hosts. Climatic variation altered soil texture, which subsequently shifted bacterial host communities and contributed to the ARG abundance. Land-use intensity may change soil resource availability or introduce ARG-carrying bacteria, thereby elevating the abundance of potential ARG hosts and indirectly increasing ARG abundance.

### Spatial patterns of ARG abundance

**Figure 5:**
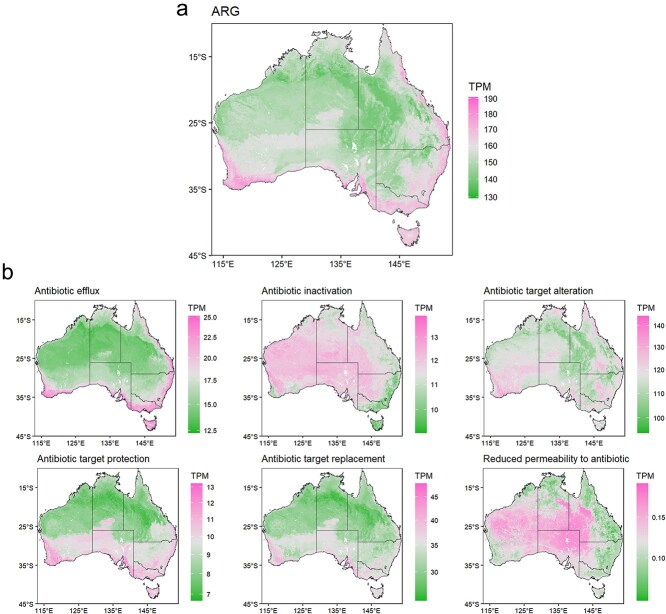
Predicted distribution of soil ARG abundance across Australia. (a) Predicted map of the total ARG abundance accoss Australia. (b) Predicted ARG abundance partitioned by resistance mechanism.

Given the pronounced spatial pattern in ARG abundance driven by climate variability and soil properties, we incorporated spatial covariates of soil and climate factors and applied a Random Forest model to predict the abundance of ARGs and their associated resistance mechanisms across Australia (Fig. 5). The resulting predictive maps (Fig. 5a) revealed clear geographical hotspots for the total ARG abundance, particularly enriched across the southwest, southeast, and northeast coastal regions. However, the ARGs assigned to specific resistance mechanisms exhibited contrasting spatial patterns (Fig. 5b). Hotspots for Antibiotic Efflux mechanisms clustered primarily along the southwest and southeast coasts, aligning with the pattern of total ARG abundance. In contrast, mechanisms involving Antibiotic Inactivation and Reduced Permeability showed hotspots predominantly localized in the central inland regions. Furthermore, resistance mechanisms related to Antibiotic Target Protection and Replacement were concentrated in the higher latitude areas. Overall, the ARG abundance model and most mechanism models achieved high predictive accuracy (*R*^2^ ranging from 0.60 to 0.86, Table S2).

## Discussion

Across Australian topsoils, our analysis shows that hydroclimatic variability is the dominant filter on ARG abundance, and that ARG hotspots emerge under climatically stable conditions, coinciding with sandy soils under agricultural use in Southwestern Australian cropland and pastureland. We found that Australian soil is predominantly dominated by mechanisms involving antibiotic target alteration and efflux pumps (Fig. 1). In this study, the most prevalent ARGs mainly confer resistance to glycopeptides and rifamycins, with target alteration exemplified by the *vanR_in_vanO_cl* cluster. Our results confirm previous studies regarding the widespread dominance of these ARG types in soils [28]. Across biomes, we further identified a set of highly prevalent genes, including *vanR_in_vanO_cl*, *rpoB2*, *rphA*, *rphB* (Table S4). The potential health risks associated with these environmental ARGs must be carefully interpreted, as their risk is shaped by both their mobilization potential via transferable genetic elements and the selection pressures implied by land-use gradients [29, 30].

### Lower climatic variability emerged as the dominant filter of soil ARGs

Across the continental transect, our analysis shows that hydroclimatic variability was the dominant filter on ARG abundance (Fig. 2a). This result aligns with the global finding that climatic seasonality is a major driver of soil resistome biogeography [7]. Beyond this general trend, we found a possible turning point at PVA of approximately 0.65 that delineates two distinct regimes: a negative association between PVA and ARG abundance when PVA < 0.65, followed by a decoupling of the relationship, with ARG levels stabilizing despite further increases in PVA (Fig. 2c).

Within the region of lowest climatic variation in Cluster A (in Southwest Australia), ARG abundance was consistently and significantly higher (TPM = 169) than in all other clusters (TPM = 141, 130, and 126, respectively) (Fig. 3). Low climatic variation, especially in precipitation, can promote more stable soil moisture, supporting microbial activity and higher microbial biomass [31, 32]. In such stable habitats, the dominant selective pressures are likely to shift from survival under abiotic extremes towards biotic interactions, consistent with classic stress–competition trade-off theory [33]. This competitive interaction may promote the maintenance and proliferation of ARGs as defensive mechanisms against antibiotic-producing competitors [34]. Furthermore, in our results, low climatic variation is strongly associated with low mean annual temperature (Fig. S2). Temperature can affect bacterial survival by regulating microbial ecological and evolutionary processes [35], with higher temperatures reducing ARG diversity [36]. Our study demonstrated that this variation-ARG relationship also holds in precipitation-limited environments.

In regions with higher precipitation variability and lower ARG abundance (Clusters C and D, in the Eastern seaboard and Northern Australia), this stability-driven accumulation mechanism appears to break down. The total abundance of ARGs is generally lower and appears to be regulated by soil properties such as pH, C:N ratio, and SOC content (Fig. S8). These soil properties may influence ARG persistence by directly altering the bioavailability of antibiotics to bacteria [37]. Mechanistically, soil pH acts as a fundamental physiological filter, reshaping bacterial community composition and may impose constraints on horizontal gene transfer (HGT) events that facilitate ARG dissemination [38, 39]. Furthermore, SOC and the C:N ratio reflect resource availability and stoichiometric constraints that can shape resistome dynamics: carbon limitation has been linked to increased antibiotic resistance, whereas higher C:N conditions may favour antibiotic production, thereby influencing the selection and persistence of ARGs [40, 41].

### Sandy and low-resource soils as unexpected ARG hotspots

Unexpectedly, ARG hotspots were concentrated in regions characterized by sandy and low-carbon soils, particularly Cluster A (in Southwest Australia) (Figs 2 and S10). Soil texture has been widely recognized as an important determinant of ARG patterns [42, 43], consistent with the differences amongst soil types in our dataset (Fig. S11). We therefore anticipated higher ARG abundance in finer-textured, carbon-rich soils, given that clay minerals can stabilize extracellular DNA [44] and higher carbon availability is often associated with greater resistome abundance [8]. One explanation would be that macro-scale climatic drivers largely override these local soil texture effects across Australian biomes. In the sandy or resource-poor environments across the clusters, the coincidence of high ARG abundance with low annual precipitation variation (Fig. S12) indicates that climatic variability may be the primary force enabling ARG persistence, effectively compensating for the lack of soil protection or nutrients. Another plausible explanation is that sandy, nutrient-poor soils impose strong environmental filtering that selects for oligotrophic taxa, such as *Actinobacteria*, which are major hosts of ARGs [45, 46]. This may raise an intriguing question for future ARG exploration: the resource-poor regions of central Australia may not be biological deserts for ARGs, but rather unexpected hotspots of resistome diversity.

### Context-dependent amplification of ARGs by agricultural land use

Land use intensity was positively associated with the ARG abundance, but this amplification effect was context-dependent, primarily in cluster A and B (in Southwest Australia and Southeast Australia) (Fig. 4). In these clusters, agricultural practices significantly elevated ARG abundance compared to natural soils: cropland increased abundance by 13.6%–15.0% and pasture drove increases of 13.3%–16.4%. Agricultural practices are recognized as a crucial pathway for antibiotics to enter soil ecosystems [47]. Anthropogenic inputs, such as fertilization and wastewater irrigation, directly introduce antibiotics and ARGs into soil ecosystems [48, 49]. Beyond fertilization, another critical factor influencing ARG difference by anthropogenic inputs is vegetation coverage (Fig. S13). Vegetation growth directly regulates the ARGs by releasing root exudates that influence antibiotic degradation, either by supplying necessary nutrients, inhibiting microbial metabolism, or altering local soil conditions [48].

Beyond direct inputs, land use may indirectly shape the resistome by altering soil physicochemical properties. Our results indicate that cropland soils exhibited a pH closer to neutral compared to natural lands in Cluster A and B (in Southwest Australia and Southeast Australia) (Fig. S14). Neutral pH is widely recognized to support higher bacterial richness [38], which may facilitate the accumulation of ARG-carrying bacteria. Besides, we also observed increased total P (TP) in cropland. The TP are likely to reflect long-term P status, which can restructure microbial biomass and community composition by shifting nutrient limitation and microbial life-history strategies, thereby changing the size and identity of the potential ARG host pool [50, 51]. Although in cluster D (in Northern Australia), ARG abundance in pasture soils was significantly lower than in natural soils, showing a decrease of ~6.11% (Fig. 4a). This may be due to the lower annual temperature in the natural soils (Fig. S15), since higher soil temperature may accelerate extracellular DNA degradation and then limit ARG persistence [52]. Taken together, these findings underscore the necessity of integrating climatic variability and land use intensity into predictive models of soil ARG distribution, particularly for forecasting ARG dynamics under future climate mitigation scenarios.

### ARG spatial hotspots and implications for surveillance

By integrating metagenomic data with spatially explicit environmental covariates, we further identify distinct spatial hotspots for different resistance mechanisms, providing a framework for targeted surveillance of environmental resistomes. Our spatial predictions reveal that ARG abundance hotspots are concentrated in south-western and eastern coastal Australia (Fig. 5a), a pattern consistent with previous global ARG maps [8]. These coastal regions are enriched in abundant organic carbon and phosphorus [53], which serve as energy sources for ARG-carrying microbes [54, 55]. This nutrient-driven enrichment does not contradict our findings in *Sandy and low-resource soils as unexpected ARG hotspots* Section regarding low-resource soils. Instead, it highlights that climatic variability provides the essential baseline for ARG accumulation in both contexts. Although stability alone allows ARGs to persist in resource-poor inland soils, in coastal regions, it enables soil nutrients to further amplify ARG loads.

Beyond total abundance, we emphasize the distinct spatial biogeography of specific resistance mechanisms (Fig. 5b). Consistent with bacterial patterns observed previously [13], ARG mechanisms exhibited coastal and latitude-related enrichment. For instance, Antibiotic Efflux mechanisms were clustered along the nutrient-rich coasts. This spatial pattern is likely driven by the bacterial hosts tracking, given that *Proteobacteria* are the primary hosts for these efflux genes [56]. As typical copiotrophs, *Proteobacteria* thrive in high-carbon coastal soils, thereby driving the accumulation of efflux genes [57]. Conversely, mechanisms involving Antibiotic Inactivation were enriched in the arid inland. This distribution is likely associated with drought-tolerant taxa such *as Actinobacteria*, which act as major reservoirs for soil ARGs [58].

Overall, based on these distinct spatial drivers, we propose a prioritized framework for soil resistome surveillance. Monitoring efforts should focus on management in ARG hotspot regions, particularly in areas with low climatic variation where intensive land use can amplify ARG loads. Adjusting land-use practices in these climatically stable regions may be especially effective for reducing ARG dissemination from soils. Although our continental-scale approach captures broad spatial patterns, it does not account for temporal or seasonal fluctuations, which may still modulate resistome profiles. Furthermore, our sampling, although capturing major anthropogenic regions, covers a relatively narrow climatic envelope which constrains the extent to which the observed relationships can be extrapolated to Australia’s full hydroclimatic range. Beyond these considerations, though this study resolves community-level environmental drivers of the soil resistome, ARGs were not formally stratified using clinical-risk frameworks. In a complementary analysis, we evaluated the subset of highest-risk ARGs [30], which are enriched for mobile and human-associated resistance genes, and found that climatic variability remained the dominant correlate (Fig. S16). Future work integrating risk-based classification, targeted assessment of clinically significant resistance pathways and measurements of specific agricultural stressors will further refine hotspot interpretation and its potential health relevance. This will provide a more informative picture of ARG dynamics, better reveal the mechanisms controlling them and improve the evidence base for pollution-risk management strategies.

## Conclusion

We established a large ARG dataset across 268 Australian topsoils and found the ARG distribution was dominantly structured by climatic gradients and modulated by land-use intensity. We further found that the climatic variability is the most crucial driver for the overall ARG abundance. By attributing ARGs to these dominant mechanisms and mapping their distributions, we establish a coherent basis for anticipating how climate variability and land-use change may propagate resistance risks. The clear delineation of climatic and land-use controls, coupled with hotspot localization, provides an evidence base for targeted surveillance and land-management interventions aimed at constraining the expansion of the soil ARG pool. Overall, these results refine the mechanistic links between climate, land use, and the topsoil resistome at the continental scale, establish a baseline for future assessments under ongoing environmental change, and provide actionable information for risk assessment and nature-positive management of agricultural and natural landscapes.

## Supplementary Material

Supplementary_materials_wrag079

## Data Availability

The datasets generated during and/or analyzed during the current study are available in the Zenodo repository, 10.5281/zenodo.18297811.
