# Supplementary material for: Macroecological processes impact Australian soil resistomes and climatically stable regions with anthropogenic activities serve as ARG hotspots
Source: ISME J. 2026 Apr 10;20(1):wrag079. doi: 10.1093/ismejo/wrag079 (PMC13157831; doi:10.1093/ismejo/wrag079)
Supplement: Supplementary_materials_wrag079 [file supplementary_materials_wrag079.docx]

Supplementary materials
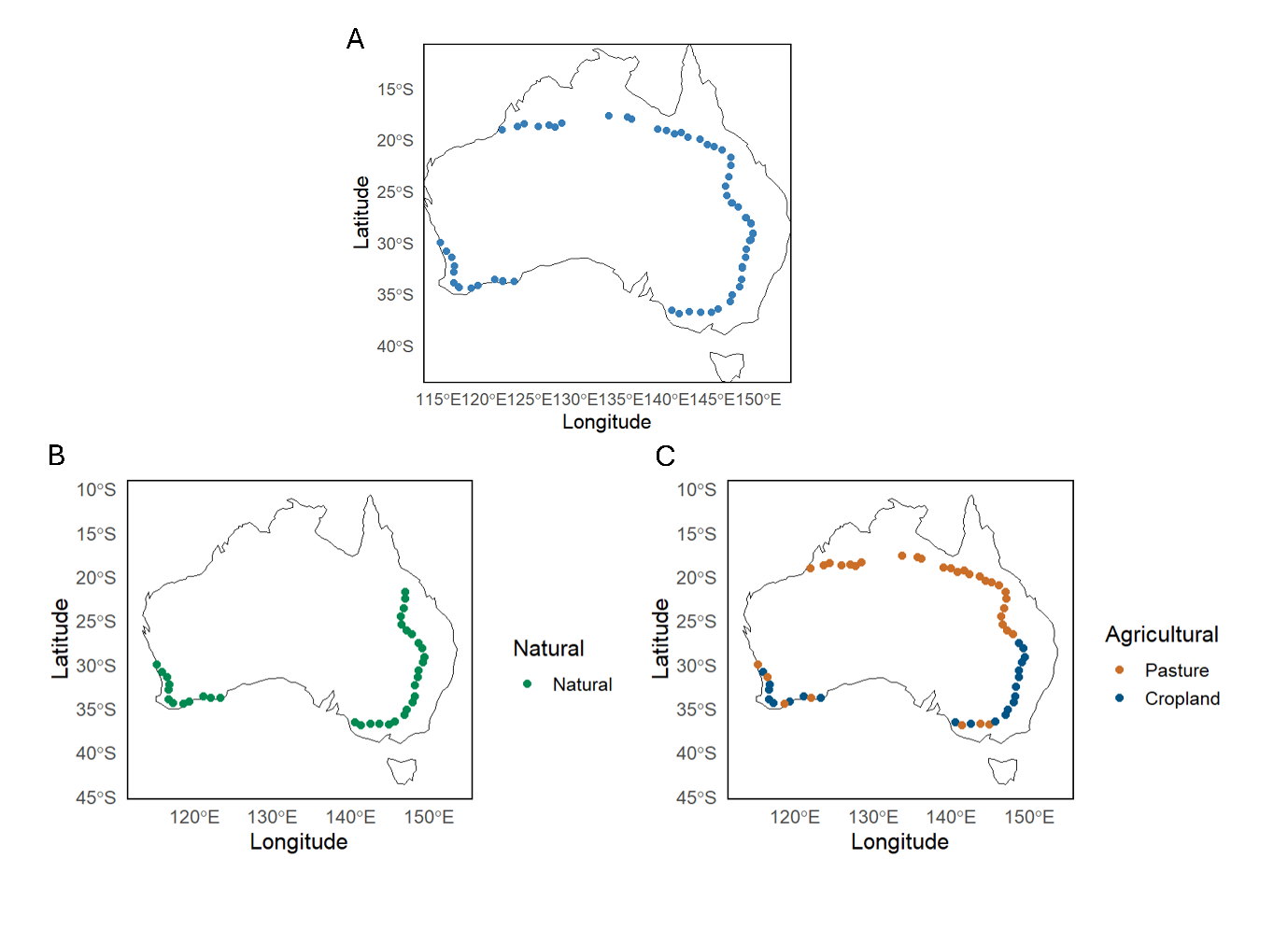


Fig.S1. Distribution of soil sampling points along the 500 - 600 mm isohyet across Australia. (A) Overview of the broad sampling regions. (B) Specific sampling locations situated on natural land. (C) Specific sampling locations situated on agricultural land, including cropland and pasture.


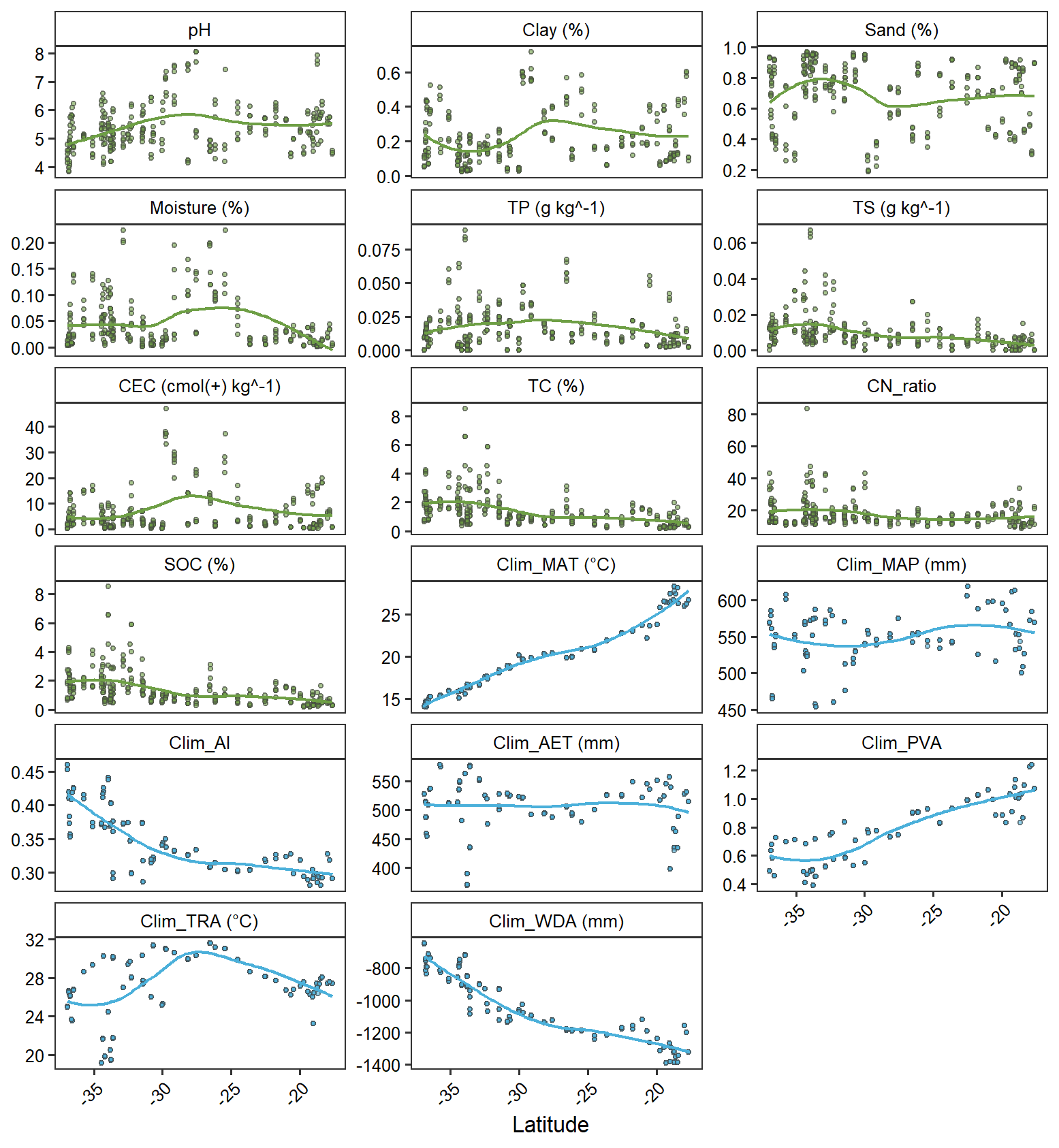


Fig.S2. Pedo-climatic factors along the latitude. TP: total phosphorus; TS: total sulfur; CEC: cation exchange capacity; TC: total carbon; CN_ratio: carbon-to-nitrogen ratio; SOC: soil organic carbon. Clim_MAT: mean annual temperature; Clim_MAP: mean annual precipitation; Clim_AI: aridity index; Clim_AET: annual actual evapotranspiration; Clim_PVA: annual precipitation variability; Clim_TRA: annual temperature range; Clim_WDA: annual atmospheric water deficit.


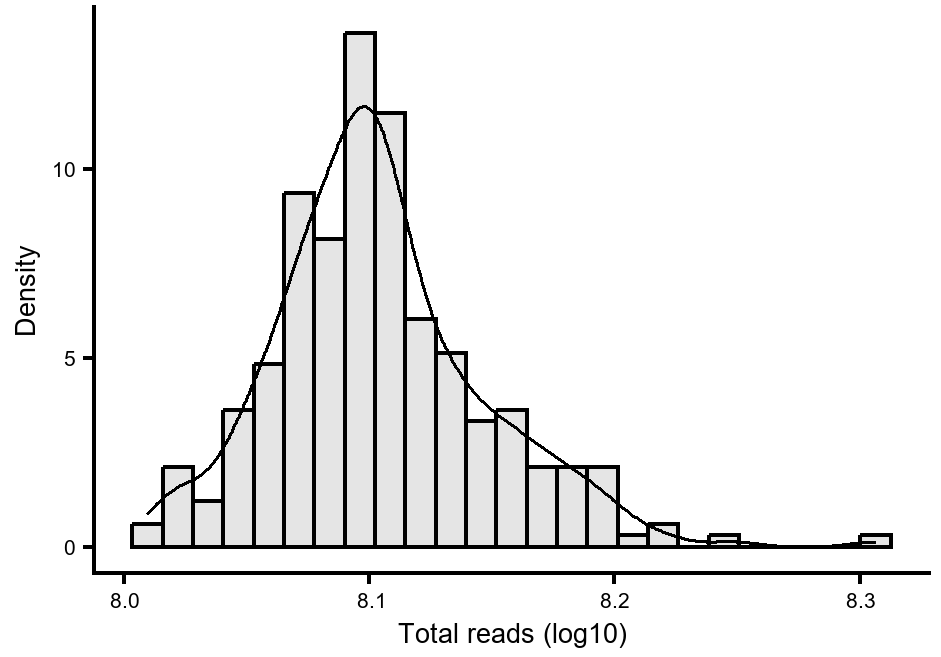


Fig.S3. Distribution of sequencing depth after quality control. Histogram (grey bars) and kernel density estimate (black line) showing the distribution of per-sample clean reads following quality filtering. The x-axis is presented on a log10 scale to facilitate visualisation of the right-skewed distribution.


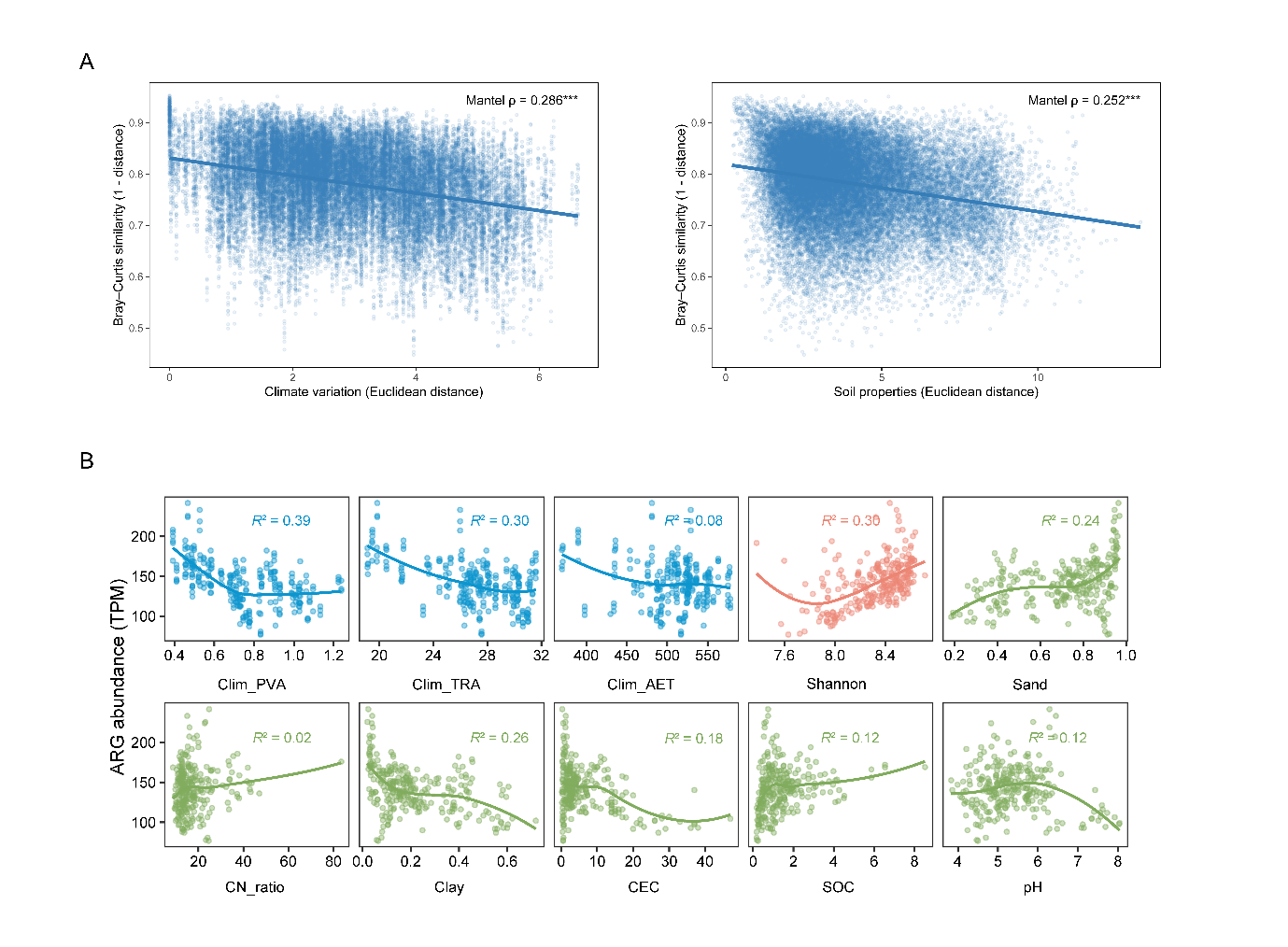


Fig.S4. Pedo-climatic controls on soil ARG abundance. (A) Relationships between ARG community similarity and pedo-climatic distance. (B) Linear relationships between environmental factors and ARG abundance. TP: total phosphorus; TS: total sulfur; CEC: cation exchange capacity; TC: total carbon; CN_ratio: carbon-to-nitrogen ratio; Clim_PVA: annual precipitation variability; Clim_TRA: annual temperature range.


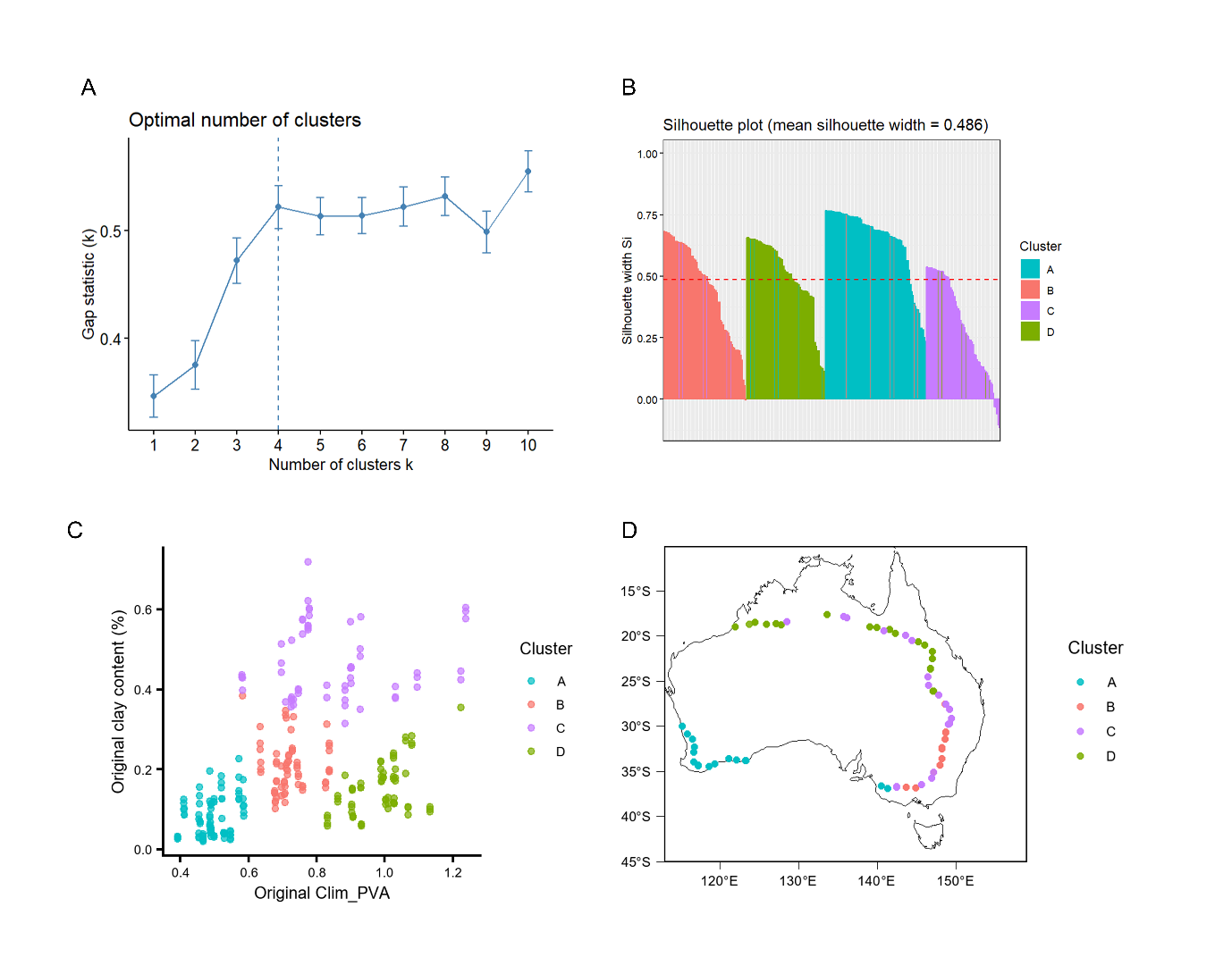


Fig.S5. Definition of pedo-climatic clusters. (A) Gap statistic across candidate cluster numbers (k) for the K-means partition based on Z-score standardised clay content and annual precipitation variability (PVA). (B) The distribution of silhouette widths across samples. (C) Relationship between PVA and clay content for the samples in different clusters. Values are shown on the original (unscaled) data for interpretability. (D) Geographic distribution of sampling sites across Australia, coloured by cluster. PVA: annual precipitation variability.


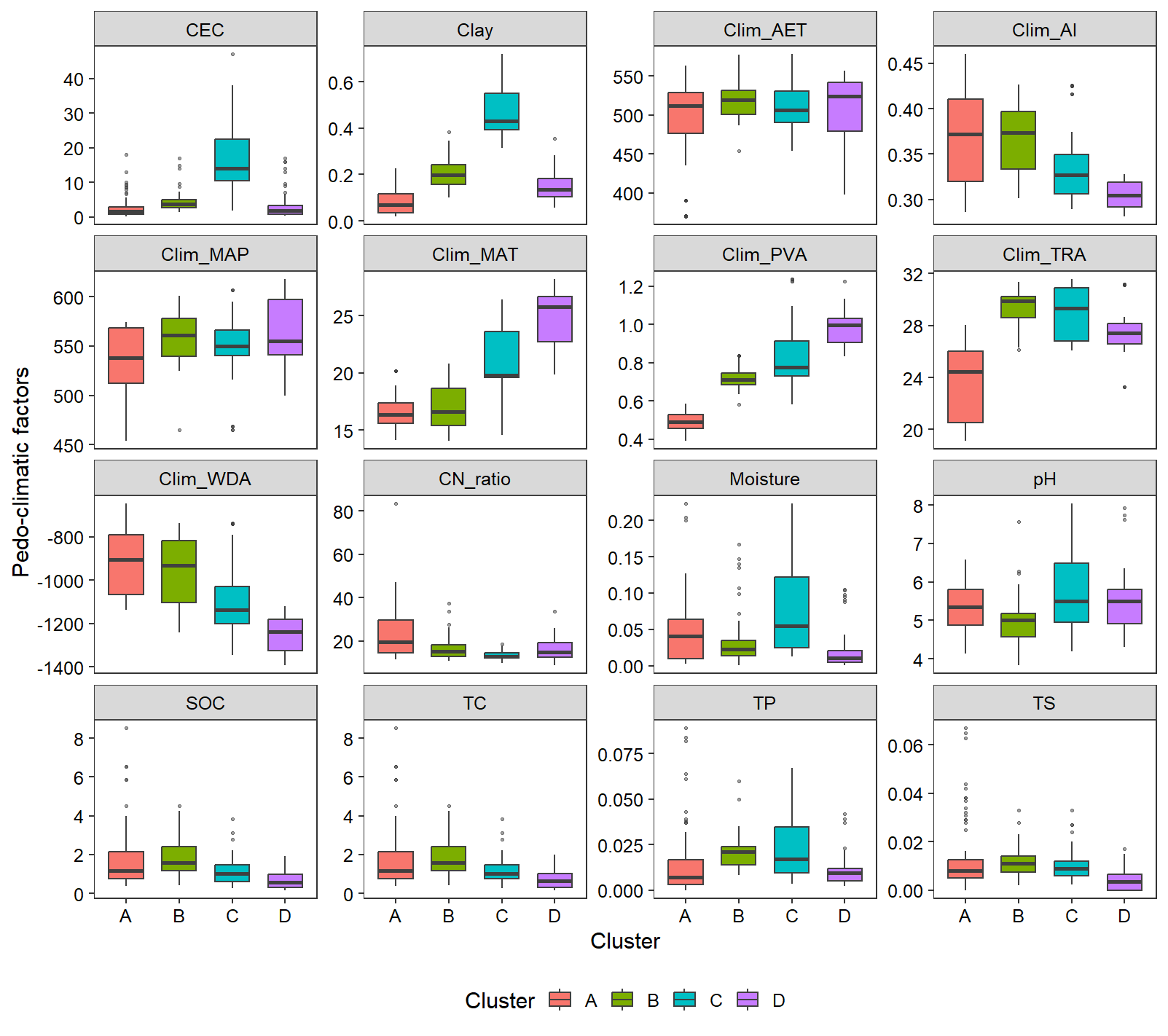


Fig.S6. Pedo-climatic values across the four clusters. TP: total phosphorus; TS: total sulfur; CEC: cation exchange capacity; TC: total carbon; CN_ratio: carbon-to-nitrogen ratio; SOC: soil organic carbon. Clim_MAT: mean annual temperature; Clim_MAP: mean annual precipitation; Clim_AI: aridity index; Clim_AET: annual actual evapotranspiration; Clim_PVA: annual precipitation variability; Clim_TRA: annual temperature range; Clim_WDA: annual atmospheric water deficit.


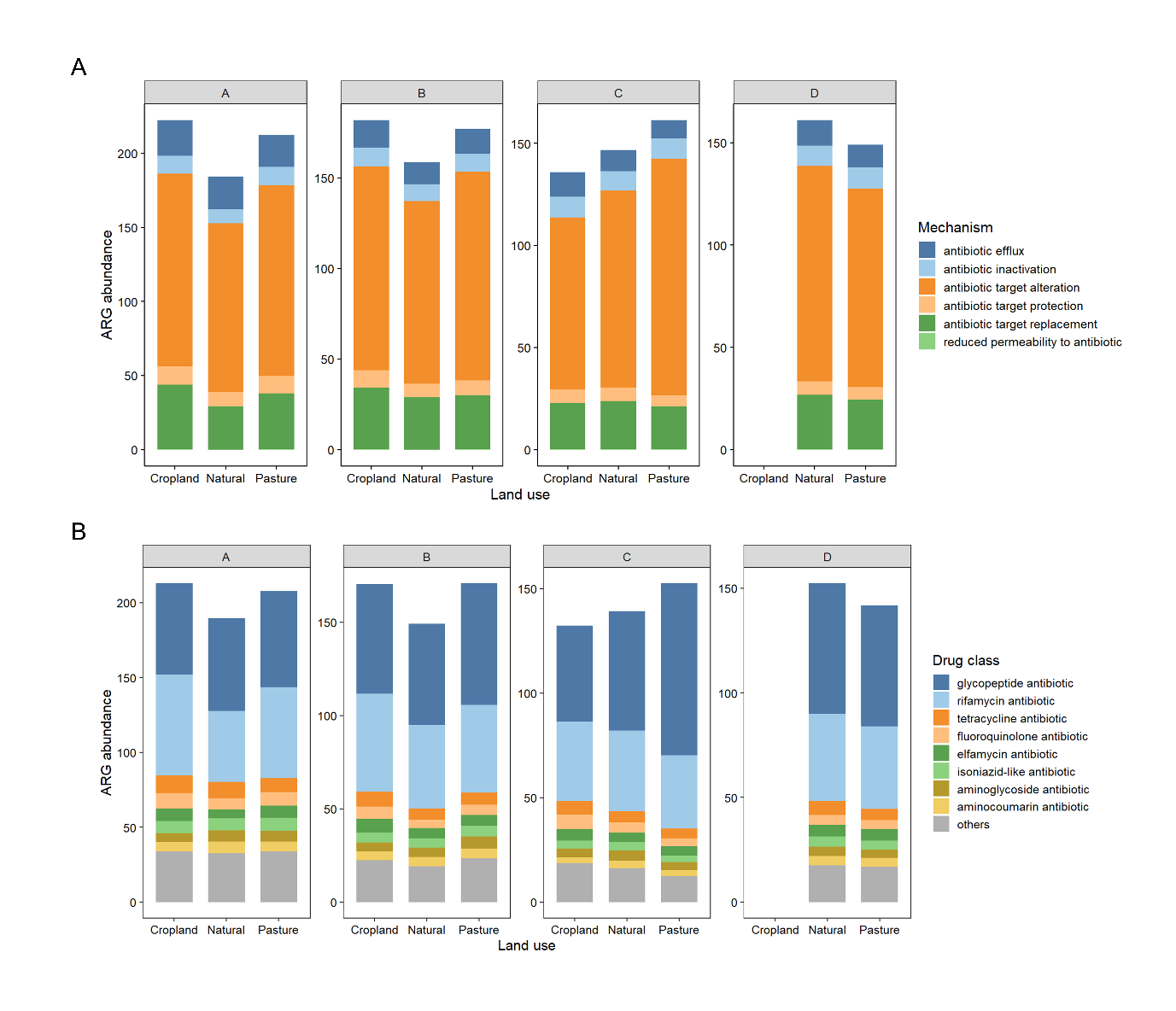


Fig. S7. Influence of land use intensity on ARG abundance across different clusters. (A) ARG abundance partitioned by resistance mechanism. (B) ARG abundance partitioned by drug class.


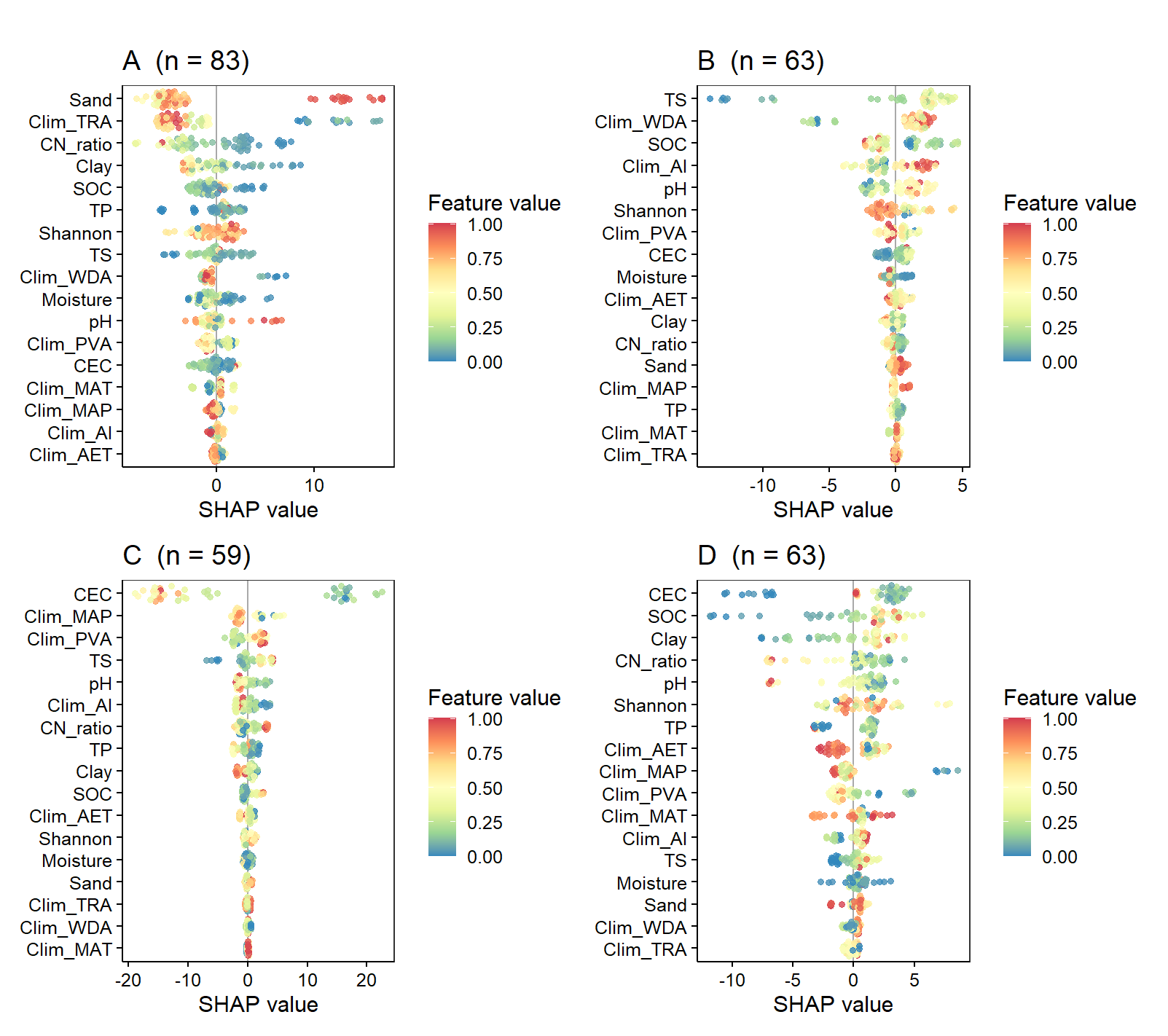


Fig. S8. Relationships between pedo-climatic factors and ARG abundance within different clusters. TP: total phosphorus; TS: total sulfur; CEC: cation exchange capacity; TC: total carbon; CN_ratio: carbon-to-nitrogen ratio; SOC: soil organic carbon. Clim_MAT: mean annual temperature; Clim_MAP: mean annual precipitation; Clim_AI: aridity index; Clim_AET: annual actual evapotranspiration; Clim_PVA: annual precipitation variability; Clim_TRA: annual temperature range; Clim_WDA: annual atmospheric water deficit.


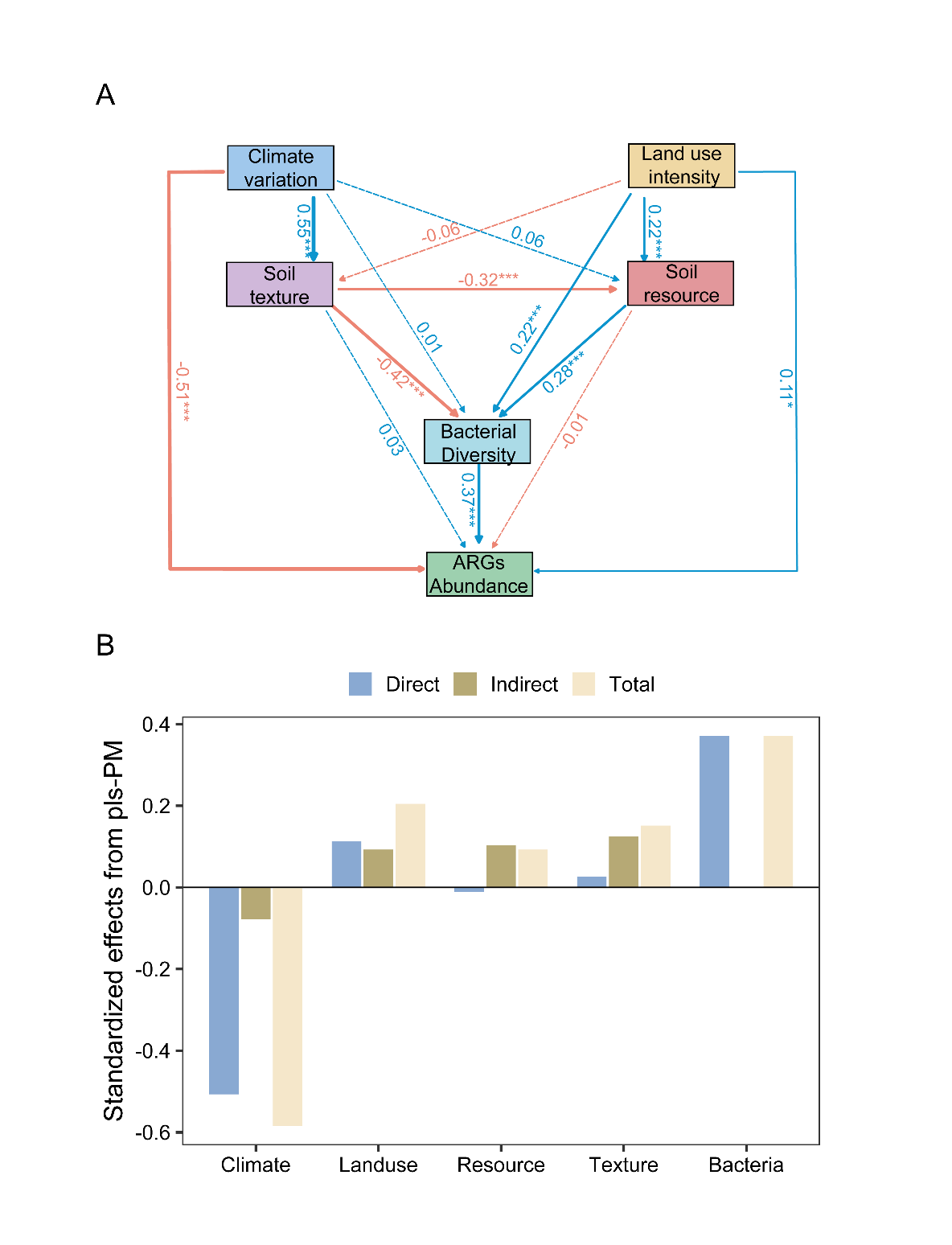


Fig. S9. Partial Least-Squares Path Model (PLS-PM) results. (A) Structural model showing pathways linking climatic variation and land-use intensity to ARG abundance. (B) Total, direct, and indirect effects of climate variation, land use intensity, soil resource, soil texture, and bacterial host Shannon index on ARG abundance, estimated based on the PLS-PM results. Blue and red arrows indicate positive and negative paths, respectively. Arrow thickness is scaled proportionally to the strength of the standardized path coefficients. Non-significant paths (*P* ≥ 0.05) are displayed as dashed arrows. Path significance levels are: **P* < 0.05; ***P* < 0.01; and ****P* < 0.001.


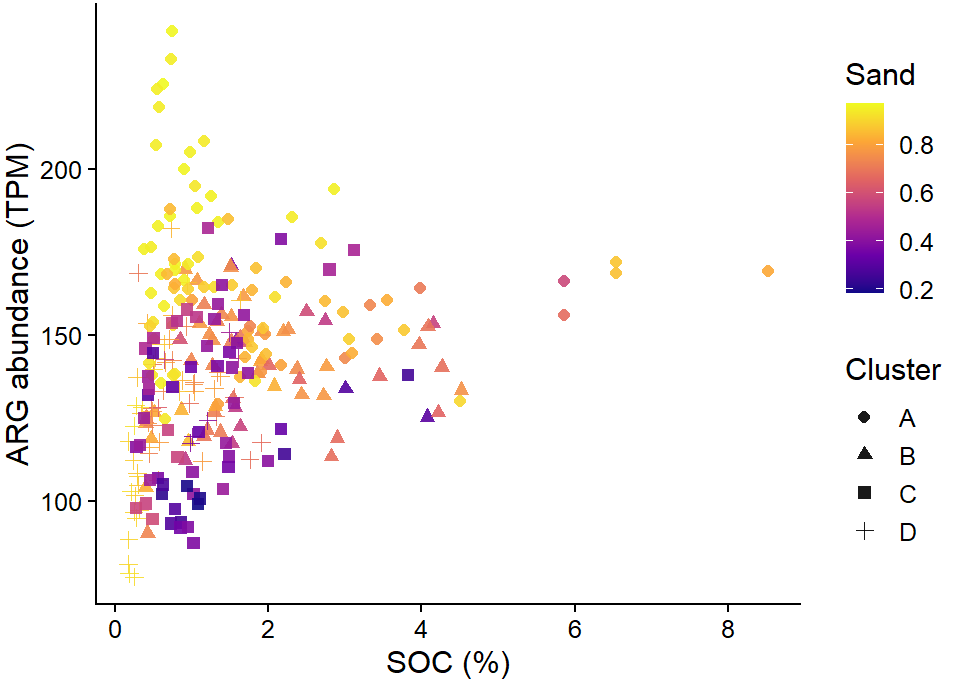


Fig.S10. Relationships between SOC content and ARG abundance. Point colour indicates sand content. Point shape indicates sample cluster (A–D).


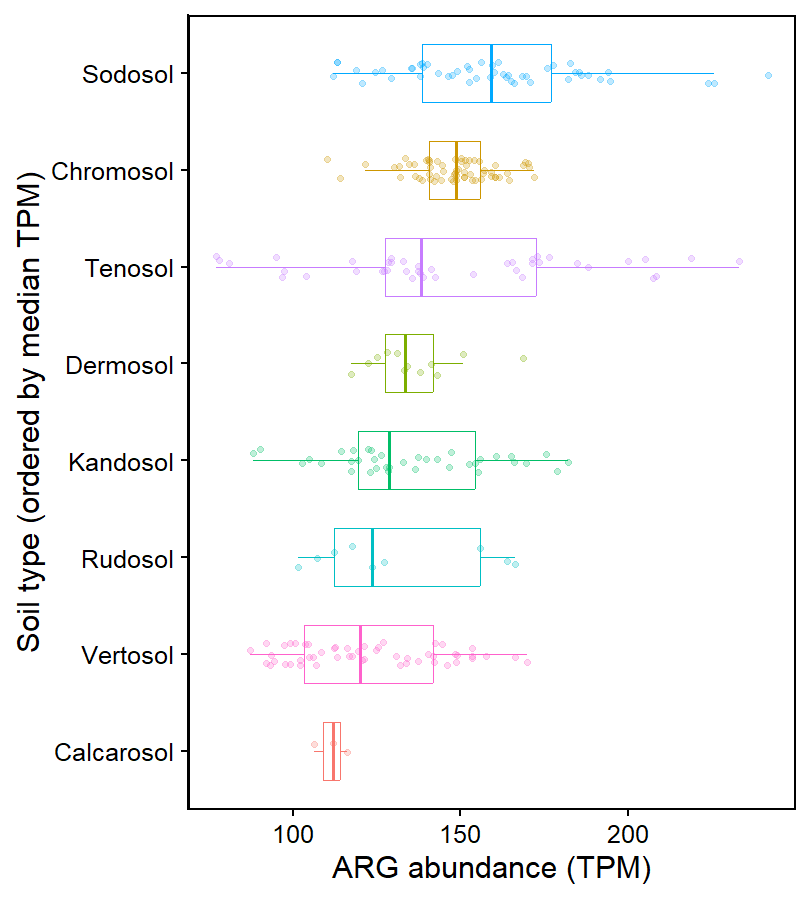


Fig.S11. ARG abundance across different soil types.


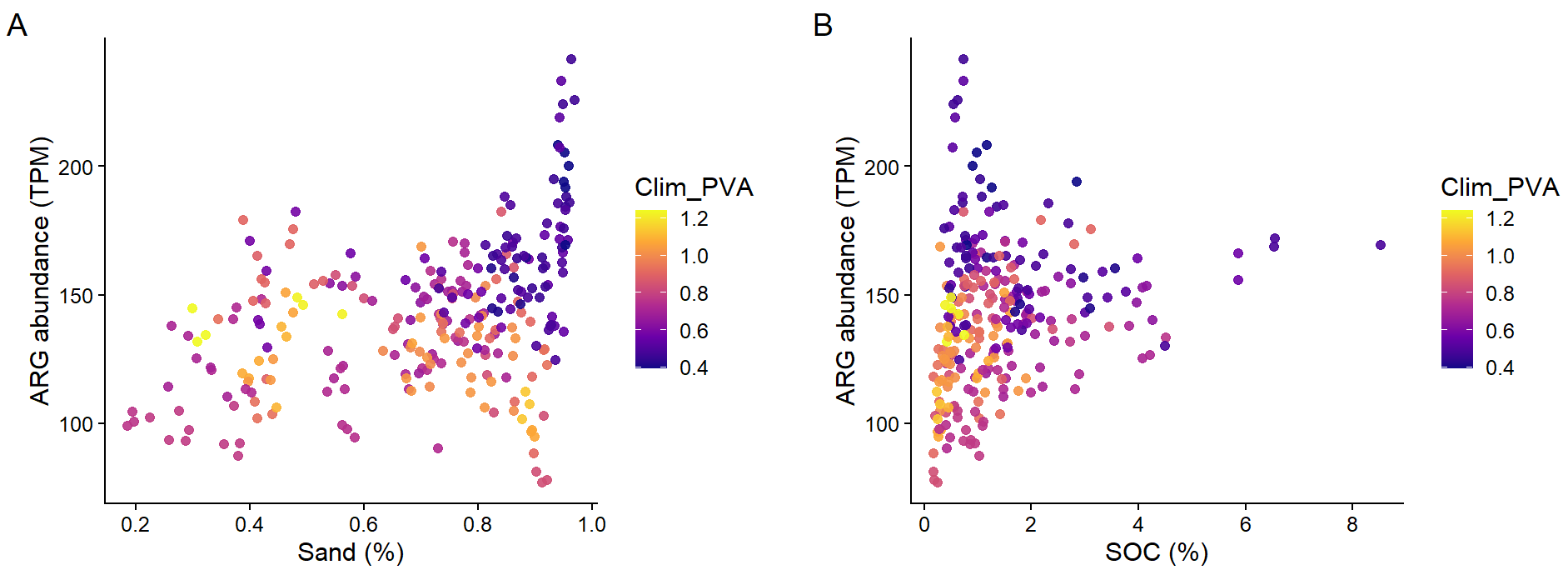


Fig.S12. Relationships between ARG abundance and soil properties across climatic variability. (A) sand content. (B) SOC content. Points are coloured by climatic variability (Clim_PVA).


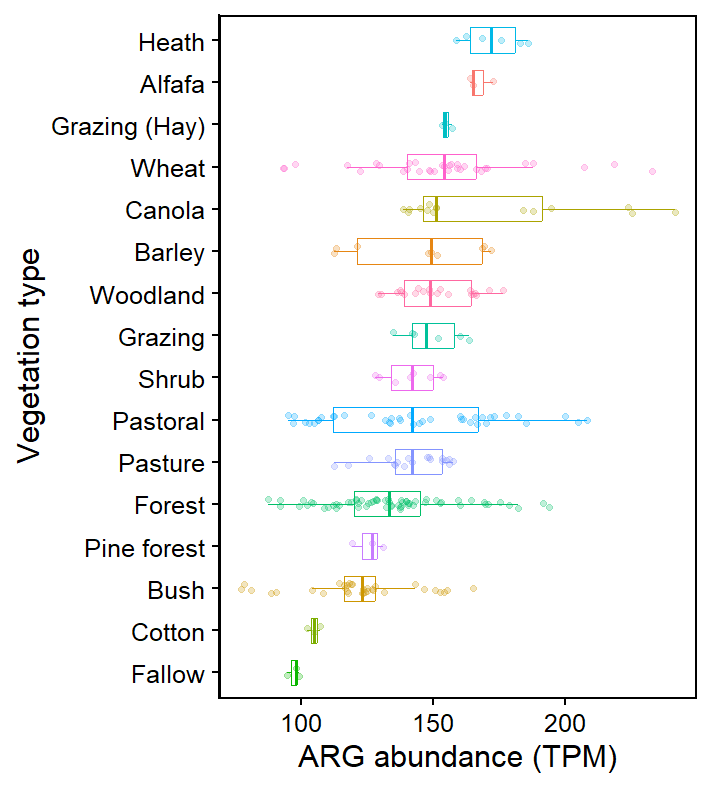


Fig.S13. ARG abundance across different vegetation types.


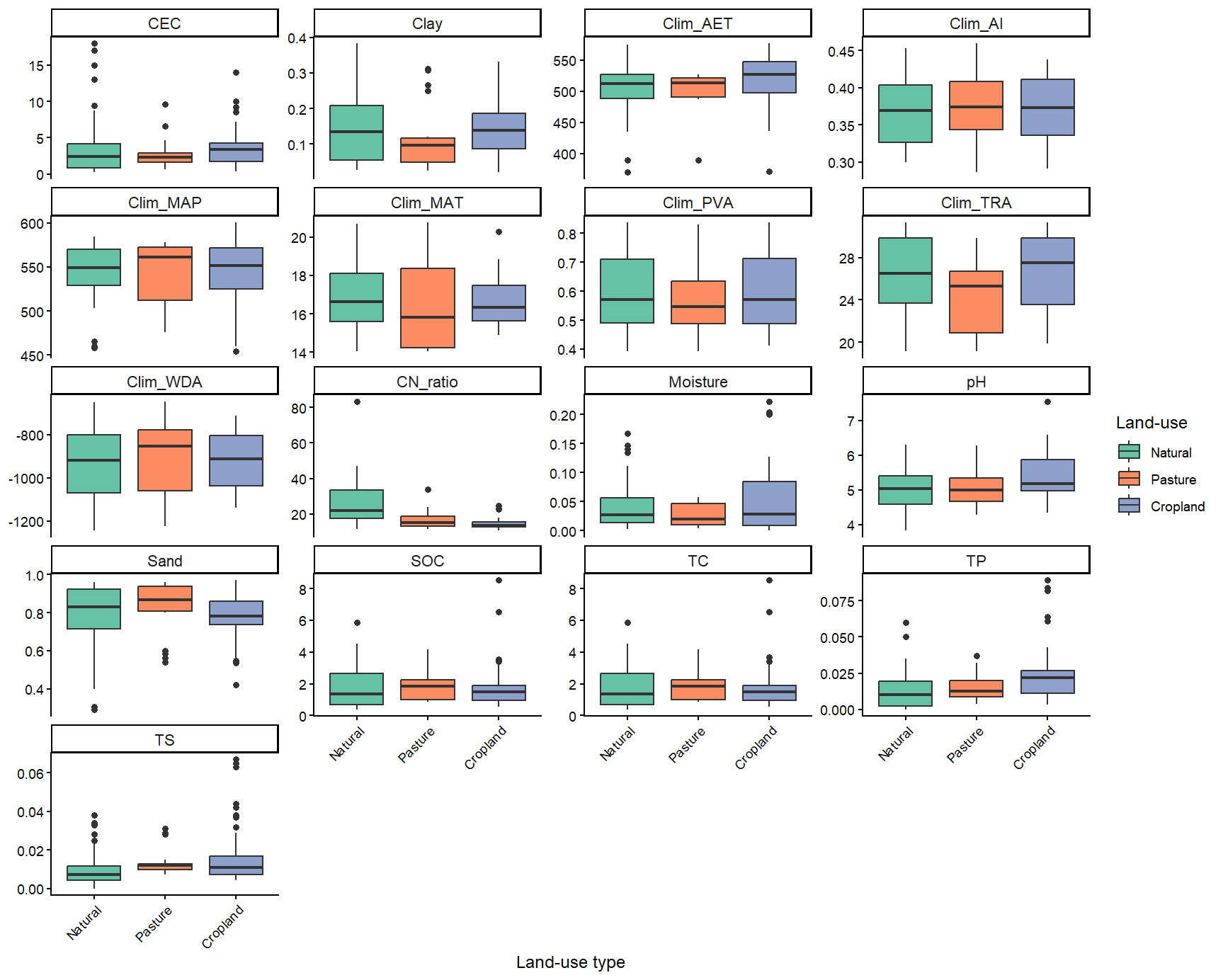


Fig.S14. Pedo-climatic properties across land-use types in clusters A and B. TP: total phosphorus; TS: total sulfur; CEC: cation exchange capacity; TC: total carbon; CN_ratio: carbon-to-nitrogen ratio; SOC: soil organic carbon. Clim_MAT: mean annual temperature; Clim_MAP: mean annual precipitation; Clim_AI: aridity index; Clim_AET: annual actual evapotranspiration; Clim_PVA: annual precipitation variability; Clim_TRA: annual temperature range; Clim_WDA: annual atmospheric water deficit.


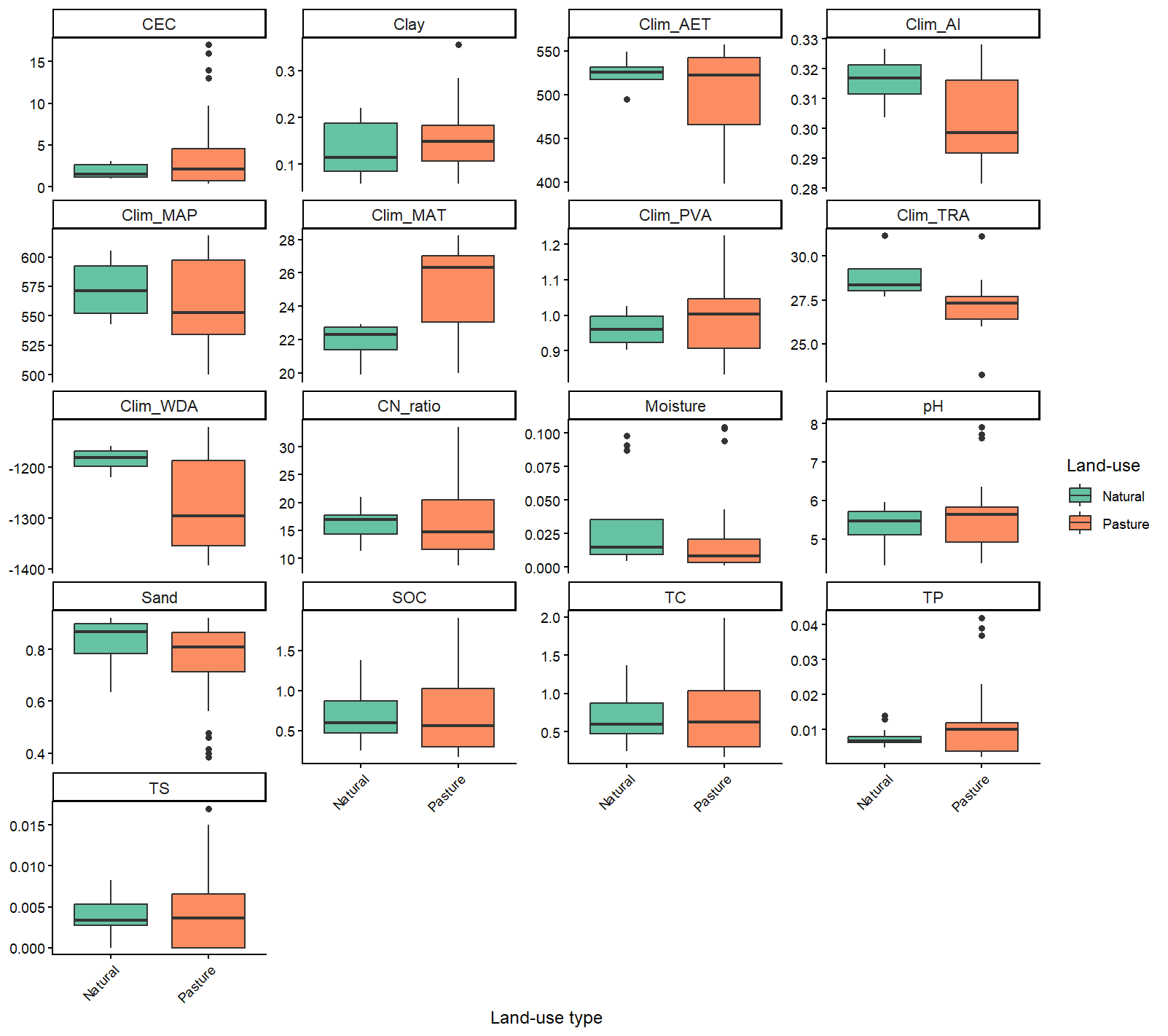


Fig.S15. Pedo-climatic properties across land-use types in cluster D. TP: total phosphorus; TS: total sulfur; CEC: cation exchange capacity; TC: total carbon; CN_ratio: carbon-to-nitrogen ratio; SOC: soil organic carbon. Clim_MAT: mean annual temperature; Clim_MAP: mean annual precipitation; Clim_AI: aridity index; Clim_AET: annual actual evapotranspiration; Clim_PVA: annual precipitation variability; Clim_TRA: annual temperature range; Clim_WDA: annual atmospheric water deficit.
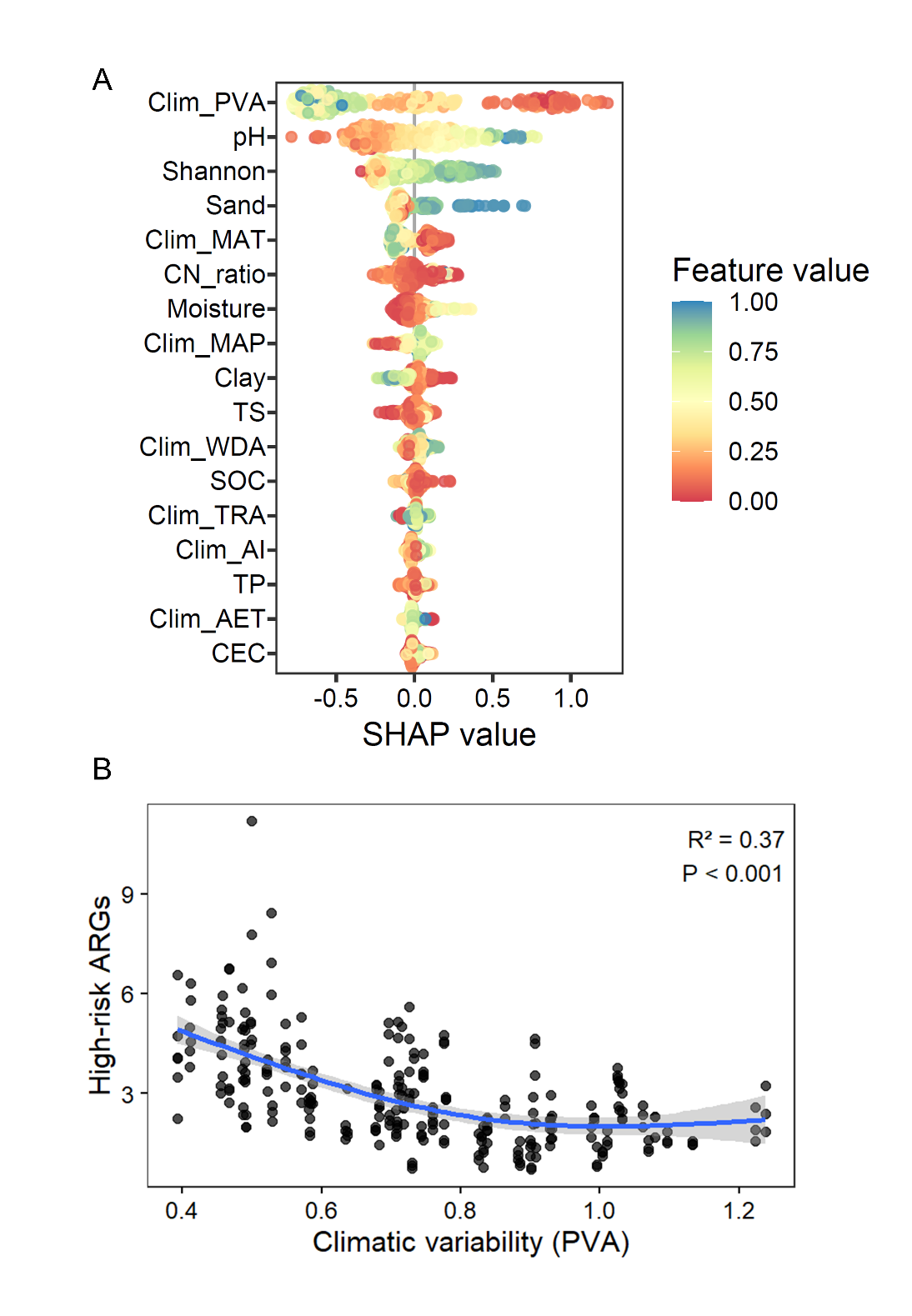


Fig.S16. Environmental drivers of high-risk ARG abundance. (A) SHAP-based feature importance and effect direction across predictors. (B) Marginal relationship between high-risk ARG abundance and climatic variability (Clim_PVA).

Table.S1: Information of the variables used for the machine learning

| Name | DataType | Attribute | Units | Description |
| --- | --- | --- | --- | --- |
| PHW_005_015_95_N_P_AU_TRN_N_20220520 | Soil | pH - Water | None | pH of a 1:5 soil water solution |
| CLY_000_005_EV_N_P_AU_TRN_N_20210902 | Soil | Clay | % | < 2 um mass fraction of the < 2 mm soil material determined using the pipette method (Version 1) |
| SND_000_005_EV_N_P_AU_TRN_N_20210902 | Soil | Sand | % | 20 um - 2 mm mass fraction of the < 2 mm soil material determined using the pipette method |
| SOC_000_005_EV_N_P_AU_TRN_N_20220727 | Soil | Organic Carbon | % | Mass fraction of carbon by weight in the < 2 mm soil material as determined by dry combustion at 900 Celsius |
| CEC_000_005_EV_N_P_AU_TRN_N_20220826 | Soil | Cation Exchange Capacity | meq/100g | Cations extracted using barium chloride (BaCl2) |
| PTO_000_005_EV_N_P_AU_NAT_C_20231101 | Soil | Total Phosphorus | % | Mass fraction of total phosphorus in the soil by weight |
| Clim_varan | DSM_Covariate | Climate | ratio | Rainfall Variability - Annual |
| Clim_TRA | DSM_Covariate | Climate | degrees celcius | Annual temperature range |
| Clim_etaaann | DSM_Covariate | Climate | mm | Average areal actual evapotranspiration - Annual |
| Relief_elev_focalrange300m_3s | DSM_Covariate | Relief | none | Relief - Elevation Range over 300 m derived from 1" SRTM DEM-S |
| Relief_slope_perc | DSM_Covariate | Relief | none | Slope derived from 1" SRTM DEM-S |
| Veg_NDVI_mean_Q1 | DSM_Covariate | Organisms | index | Long-term average Normalized Difference Vegetation Index (NDVI) |
| Veg_NDVI_mean_Q2 | DSM_Covariate | Organisms | index | Long-term average Normalized Difference Vegetation Index (NDVI) |
| Veg_NDVI_mean_Q3 | DSM_Covariate | Organisms | index | Long-term average Normalized Difference Vegetation Index (NDVI) |
| Veg_NDVI_mean_Q4 | DSM_Covariate | Organisms | index | Long-term average Normalized Difference Vegetation Index (NDVI) |

Table.S2. The performance of the QRF model (ME = mean error, RMSE = root mean square error, ρ_C_ = concordance correlation coefficient).

|  | ME | RMSE | *R*^2^ | ρ_C_ |
| --- | --- | --- | --- | --- |
| ARGs | -0.33 | 11.77 | 0.82 | 0.89 |
| Antibiotic efflux | -0.03 | 2.42 | 0.86 | 0.92 |
| Antibiotic inactivation | -0.01 | 1.18 | 0.60 | 0.72 |
| Antibiotic target alteration | 0.02 | 10.38 | 0.77 | 0.85 |
| Antibiotic target protection | 0.05 | 1.33 | 0.81 | 0.89 |
| Antibiotic target replacement | 0.10 | 4.39 | 0.77 | 0.86 |
| Reduced permeability to antibiotic | 0.01 | 0.07 | 0.05 | 0.26 |

Table.S3: Key R packages used in the statistical analyses.

| Package | Version | References |
| --- | --- | --- |
| rnaturalearth | 1.0.1 | South and South (2017) |
| sf | 1.0.21 | Pebesma (2018) |
| terra | 1.8.93 | Hijmans et al. (2022) |
| vegan | 2.7.2 | Oksanen et al. (2013) |
| xgboost | 3.1.3.1 | Chen et al. (2020) |

Table.S4: Mean ARG abundance of the top 10 ARGs

| Gene | OccurCount | OccurFreq | MeanTPM | Drug Class | Resistance Mechanism |
| --- | --- | --- | --- | --- | --- |
| vanR_in_vanO_cl | 268 | 1 | 53.22 | glycopeptide antibiotic | antibiotic target alteration |
| rpoB2 | 268 | 1 | 18.90 | rifamycin antibiotic | antibiotic target alteration; antibiotic target replacement |
| *Mtub_rpoB_RIF* | 268 | 1 | 8.62 | rifamycin antibiotic | antibiotic target alteration; antibiotic target replacement |
| Escherichia coli EF-Tu mutants conferring resistance to Pulvomycin | 268 | 1 | 4.93 | rifamycin antibiotic | antibiotic target alteration |
| HelR | 268 | 1 | 4.84 | rifamycin antibiotic | antibiotic target protection |
| rphA | 268 | 1 | 4.26 | rifamycin antibiotic | antibiotic inactivation |
| Mycobacterium tuberculosis rpsL mutations conferring resistance to Streptomycin | 268 | 1 | 3.51 | aminoglycoside antibiotic | antibiotic target alteration |
| rphB | 268 | 1 | 3.04 | rifamycin antibiotic | antibiotic inactivation |
| Mycobacterium tuberculosis katG mutations conferring resistance to isoniazid | 268 | 1 | 2.93 | isoniazid-like antibiotic | antibiotic target alteration |
| vanS gene in vanO cluster | 268 | 1 | 2.14 | glycopeptide antibiotic | antibiotic target alteration |

References:

1. South, A.,South, M. A. R Package: rnaturalearth. World Map Data from Natural Earth, Version. 2017;1.

2. Pebesma, E. Simple features for R: standardized support for spatial vector data. 2018.

3. Hijmans, R. J., Bivand, R., Forner, K. et al. Package ‘terra’. Maintainer: Vienna, Austria. 2022;384.

4. Oksanen, J., Blanchet, F. G., Kindt, R. et al. Package ‘vegan’. Community ecology package, version. 2013;2:1-295.

5. Chen, J., Zhao, F., Sun, Y. et al. Improved XGBoost model based on genetic algorithm. International Journal of Computer Applications in Technology. 2020;62:240-245.
